# Association of lymph node involvement with the prognosis of pathological T1 invasive non-small cell lung cancer

**DOI:** 10.1186/s12957-017-1098-3

**Published:** 2017-03-17

**Authors:** Yong-Kui Zhang, Zheng-da Chai, Lin-lin Tan, Zhao-yu Wang, Zhi-jun Chen, Han-Bo Le, Wang-Yu Zhu

**Affiliations:** 1 Department of Cardio-Thoracic Surgery, and Lung Cancer Research Center, Zhoushan Hospital of Wenzhou Medical University, Zhoushan, Zhejiang 316021 China; 2 Lung Cancer Research Center, Zhoushan Hospital of Wenzhou Medical University, Zhoushan, Zhejiang 316021 China; 3 Zhoushan Hospital of Wenzhou Medical University, Zhoushan, Zhejiang 316021 China

**Keywords:** NSCLC, Lymph node metastasis, Pathological T1 disease, Prognosis

## Abstract

**Background:**

Lymph node involvement could help to predict the prognosis of pathological T1 (pT1, diameters of ≤3 cm) non-small cell lung cancer (NSCLC). This study assessed the clinicopathological factors and associated lymph node involvement in invasive lung adenocarcinoma (IAC) and squamous cell lung cancer (SCC) and the overall and disease-free survival associated with these factors.

**Methods:**

Three hundred and twenty-five patients with pathological T1 NSCLC (253 IAC and 72 SCC) were retrospectively analyzed from a pool of 1094 primary lung cancer patients. The data were assessed using multiple logistic regression, Kaplan-Meier curves and multivariable analyses.

**Results:**

Among patients with a ≤30-mm tumor lesion (*N* = 325), N1 and N2 lymph node involvement was found in 28 (8.6%) and 34 (10.4%) patients, respectively. Lymph node metastasis occurred in 13.0% (33/253) of pT1 IAC patients and 40.3% (29/72) of SCC patients. Carcinoembryonic antigen (CEA) levels, SCC by histology, and tumor lesions larger than 1.0 cm were associated with lymph node involvement (*P* < 0.0001, <0.0001, and 0.048, respectively). In IAC patients, negative lymph nodes were associated with better overall survival compared with lymph node-positive ones (*P* = 0.021). No significant difference was observed in SCC patients regardless of lymph node status (*P* = 0.40). Multivariable Cox analysis revealed that lymph node involvement was an independent prognostic predictor of overall IAC patient survival (*P* = 0.041), but not of SCC patient survival (*P* = 0.470). Chemotherapy was administered to 72.2% (52/72) of SCC patients, a significantly higher rate when compared with that of IAC patients (42.3%, 107/253).

**Conclusions:**

Lymph node metastasis was inversely associated with the overall survival of IAP patients, but not with the survival of SCC patients. Patients with pT1 SCC exhibited a significantly higher rate of lymph node involvement when compared with IAC patients. Thus, a systematic lymph node dissection should be performed in pT1 IAC patients, especially in patients with IAC larger than 1.0 cm, for additional treatment selections to improve survival.

## Background

Lung cancer is one of the leading causes of cancer-related mortality in the world [1, 2]. Cancer metastasis (e.g., lymph node metastasis or distant metastasis to other organs) significantly contributes to lung cancer patient death [3, 4]. Non-small cell lung cancer (NSCLC) accounts for up to 85% of all lung cancer cases. The vast majority of diagnosed NSCLCs are adenocarcinomas (ADCs) or squamous cell carcinomas (SCCs), and the incidence of lung ADC has increased rapidly, becoming the most common histological subtype [5, 6].

The effect of clinicopathological characteristics on lung cancer lymph node metastasis in patients with pathological T1 (pT1) NSCLC remains the subject of debate, including histological subtype, tumor localization, and pleural and vascular invasion. Several previous studies reported that lung adenocarcinoma in situ (AIS) and minimally invasive adenocarcinoma (MIA) exhibited 100% 5-year disease-free survival (DFS) after lung resection [7, 8]. In contrast, invasive adenocarcinoma (IAC) contributes significantly to lymph node metastasis [9]. Thus, the data on lymph node metastasis from IAC should be evaluated regardless of AIS and MIA. Previous studies [10, 11] demonstrated that there was no difference in lymph node metastasis between SCC and lung adenocarcinoma, but not IAC in pT1 NSCLC. Therefore, the difference of lymph node metastasis from IAC and SCC should be fully evaluated with a tumor size less than 30 mm (stage T1). Yasuhiro et al. [12] reported that clinicopathological factors, such as tumor size, maximum standardized uptake value (SUV), and serum tumor markers, may not predict lymph node metastasis in patients with lung pT1 SCC [10]. Nevertheless, others have indicated that clinicopathological factors can be useful aids to predict the lymph node metastasis and the lymph node involvement-related prognosis in early stage NSCLC patients [11, 13]. Thus, further study is needed to assess and evaluate whether other clinicopathological characteristics could be useful predictors for lung cancer lymph node metastasis and to determine whether lymph node involvement is associated with survival of such lung cancer patients.

Thus, in this study, we identified and assessed clinicopathological factors potentially associated with lymph node metastasis. Furthermore, we characterized the prognosis of pT1 IAC and SCC patients with lymph node involvement.

## Methods

### Study population

In this study, we retrospectively reviewed and analyzed 1094 primary lung cancer cases that had been treated via surgical resection at Zhoushan Hospital (Zhejiang, China) between January 2007 and December 2014. All patients received routine or contrast-enhanced chest computed tomography (CT) scans (Sensation 16, Siemens, Erlangen, Germany) prior to surgery. Preoperative cardiopulmonary tests, abdominal CT or abdominal ultrasonography imaging, brain magnetic resonance or brain CT imaging, and bone scanning were also performed on all patients. After surgery, NSCLC diagnosis was confirmed histologically in accordance with the World Health Organization classification system by two pathologists. Lung adenocarcinoma was classified using the new TNM-7 version IASLC/ATS/ERS staging system [6]. All patients received systematic hilar and mediastinal lymphadenectomies according to the 2011 National Comprehensive Cancer Network (NCCN) guidelines. Lymph node N stages were determined by reviewing intraoperative frozen sections and post-surgical tissue sections of resected lymph node tissues collected during lobectomy or limited resection (segment or wedge). No patients received preoperative chemotherapy, radiotherapy, or chemoradiotherapy.

We also collected relevant clinicopathological factors, including age, gender, lymphatic and vascular vessel invasion, bronchial invasion, preoperative serum carcinoembryonic antigen (CEA) levels, and the performance of relevant surgical procedures. CEA levels >5.0 IU/Ml were assessed as positive, as defined by the assay kit (Beckman Coulter, USA). Eighty-four patients were excluded from this study due to the presence of multiple lung lesions or other exclusion factors. Patients who met exclusion criteria included (i) 17 patients with large cell lung cancer, 10 with adenosquamous carcinoma, 6 with pulmonary sarcomatoid carcinoma, 6 with lymphoepithelioma-like carcinoma, 1 with lung pleomorphic carcinoma, and 15 with neuroendocrine tumors, (ii) 305 NSCLC patients with pathological T2–T4 stage diseases, and (iii) 325 patients with a subtype of AAH, AIS, or MIA without any lymph node metastases [7, 8]. The remaining 325 patients with pT1 (253 with IAC and 72 with SCC) were included in this study. Among the patients included in the study, there were 226 with IA stage, 1 with IB stage, 24 with IIA stage, 1 with IIB stage, and 33 with IIIA stage. This study was approved by the Ethics Committee of Zhoushan Hospital of Wenzhou Medical University (Zhoushan, Zhejiang, China). All participants, or their next kin, provided written informed consent before study enrollment.

### Adjuvant chemotherapy

Platinum-based adjuvant chemotherapy was given to 157 patients with risk factors (such as lymph node metastasis, pleural invasion, and poor tumor differentiation) for 4 to 6 weeks after surgery. No mortality occurred in these 157 patients after adjuvant chemotherapy.

### Follow-up

Follow-up examinations were conducted for all patients, and the follow-up visit occurred in our outpatient clinic at 3-month intervals for the first year and 6-month intervals thereafter. CT scans and serum CEA levels were used to assess tumor recurrence. Follow-up duration ranged from 2 to 102 months, with a mean of 36 months. The last follow-up visit occurred in the month of June in 2015. The study endpoint was patient death, or the final follow-up visit. Overall survival was defined as the time from surgery to death, or to the final follow-up visit. The length of survival was defined as the number of months from the date of surgical resection to the date of patient death, or the final follow-up visit.

### Statistical analysis

All statistical analyses were performed using the Statistical Package for Social Sciences software, version 17.0 (SPSS, Inc., Chicago, IL, USA). Pearson’s chi-squared test or Fisher’s exact test was used to estimate the statistical significance between the categorized groups. The Kaplan-Meier curves were used to assess patient survival. Multivariable analyses of prognostic factors were performed using Cox’s proportional hazard regression model. All factors with univariate significance *P* values less than 0.05 were entered into a multivariable Cox model to estimate overall survival and lung cancer-specific survival. All statistical tests were two-sided and *P* value ≤ 0.05 was considered statistically significant.

## Results

### Patient characteristics

Three hundred and twenty-five eligible patients with pathological T1 NSCLC were included in this study, including 253 (77.8%) IAC and 72 (22.2%) SCC patients. Clinicopathological characteristics are summarized in Table 1. In brief, there were 187 males and 138 females with mean age of 62 years (±9 years, range 33 to 83 years). Two hundred and eighty-eight patients (88.6%) underwent lobectomy, and 37 patients (11.4%) underwent limited resection of tumor lesions. Overall, we collected 3456 lymph nodes from these 325 patients. In 874 lymph nodes from 62 patients, 197 were identified to have tumor metastasis [197/874 (22.5%)]. Among these 62 patients, 28 (8.6%) and 34 (10.8%) had N1 and N2 lymph node involvement, respectively. The 34 patients with N2 lymph node involvement had both N1 and N2 positive lymph nodes in 19 (5.8%) and 15 (4.6%) patients who exhibited nodal skip metastasis, respectively (Table 1).

**Table 1:** Lymph node involvement and clinicopathological characteristics of all pT1 patients, *n* (%)

Characteristics	pN0 (*n* = 263)	pN1 (*n* = 28)	pN1+N2 (*n* = 19)	pN2 (*n*=15)	*P* value
Age (mean), yrs.	62 ± 9	61 ± 7	59 ± 9	58 ± 7	
≤60	101 (31.1)	12 (3.7)	7 (2.2)	10 (3.1)	0.185
>60	162 (49.8)	16 (4.9)	12 (3.7)	5 (1.5)	
Gender					
Male	145 (44.6)	22 (6.8)^a^	14 (4.3)	6 (1.8)	0.020
Female	118 (36.3)	6 (1.8)	5 (1.5)	9 (2.8)	
Tobacco smoking history					
Never	147 (45.2)	18 (5.5)	6 (1.8)^b^	9 (2.8)	0.044
Ever/current	116 (35.7)	10 (3.1)	13 (4.0)	6 (1.8)	
CEA level (*n* = 395)					
<5.0 IU/mL	216 (66.5)^c^	16 (4.9) ^c^	9 (2.8)	9 (2.8)	<0.0001
≥5.0 IU/mL	47 (14.5)	12 (3.7)	10 (3.1)	6 (1.8)	
Tumor location					
Upper lobe	154 (47.4)	12 (3.7)	10 (3.1)	10 (3.1)	0.150
Middle lobe	18 (5.5)	2 (0.6)	1 (0.3)	2 (0.6)	
Lower lobe	87 (26.8)	12 (3.7)	6 (1.8)	2 (0.6)	
Middle-lower lobe	4 (1.2)	2 (0.6)	2 (0.6)	1 (0.3)	
Type of surgery					
Lobectomy	235 (72.3)	23 (7.1)	17 (5.2)	13 (4.0)	0.720
Limited resection	28 (8.6)	5 (1.5)	2 (0.6)	2 (0.6)	
Adjuvant chemotherapy					
No	166 (51.1)^d^	0^d^	0^d^	0^d^	<0.0001
Yes	97 (29.8)	28 (8.6)	19 (5.8)	15 (4.6)	
Tumor size, cm					
≤1.0	28 (8.6)^e^	0^e^	0	0	0.021
1.0–≤2.0	153 (47.1)	12 (3.7)	10 (3.1)	7 (2.2)	
2.0–≤3.0	82 (25.2)	16 (4.9)	9 (2.8)	8 (2.5)	
Histology					
IAC	220 (67.7)^f^	12 (3.7)^f^	10 (3.1)^f^	11 (3.4)^f^	<0.0001
SCC	43 (13.2)	16 (4.9)	9 (2.8)	4 (1.2)	

*CEA* carcinoembryonic antigen, *IAC* invasive lung adenocarcinoma, *SCC* squamous cell lung cancer

^a^Male vs*.* female

^b^never smoked tobacco vs*.* ever/current

^c^CEA level <5.0 IU/mL vs. ≥5.0 IU/mL

^d^no adjuvant chemotherapy vs*.* adjuvant chemotherapy

^e^tumor size ≤1.0 vs. 1.0–≤2.0 vs*.* 2.0–≤3.0

^f^IAC vs. SCC

Among the 325 patients, 28 (8.6%) had primary pathological tumor lesions measuring ≤1.0 cm, whereas 182 (56.0%) had tumor lesions between 1.0 and 2.0 cm and 115 (35.4%) had tumor lesions between 2.0 and 3.0 cm. Patients with subcentimeter-sized tumor lesions did not exhibit lymph node metastasis. Pathologically, positive lymph nodes were present in 29 (15.9%) patients with tumor lesions between 1.0 and 2.0 cm and 33 (28.7%) of the patients with tumor lesions between 2.0 and 3.0 cm. Lymph node involvement was observed in 18.3% (33/180) of the non-smokers and 20.0% (29/145) of the smokers. Lymph node metastasis occurred in 13.0% (33/253) of pT1 IAC patients and 40.3% (29/72) of SCC patients. Significant differences in lymph node involvement (pN1, pN1+N2 or pN2) were observed in pT1 patients when the patients were segregated according to sex, preoperative CEA levels, pathological tumor size, and histology (*P* = 0.020, <0.0001, 0.021 and <0.0001, respectively). However, no statistical differences were observed when age, tumor location, and surgical procedures were analyzed (*P* = 0.185, 0.150, and 0.720, respectively).

Additionally, differences were observed in IAC and SCC patients with lymph node involvement and with a tumor lesion smaller than 30 mm. In IAC patients, preoperational CEA levels and pathological tumor size were associated with lymph node involvement (*P* < 0.0001 and 0.015, respectively). No association was observed between lymph node involvement and clinicopathological data in small-sized SCC (Table 2).

**Table 2:** Lymph node involvement and clinicopathological characteristics of pT1 IAC and SCC patients, *n* (%)

Histology	IAC		*P* value	SCC		*P* value
	pN0 (*n* = 220)	pN (+) (*n* = 33)		pN0 (*n* = 43)	pN (+) (*n* = 29)	
Age (mean), yrs.						
≤60	90 (40.9)	17 (51.5)	0.263	11 (25.6)	12 (41.4)	0.201
>60	130 (59.1)	16 (48.5)		32 (74.4)	17 (58.6)	
Gender						
Male	104 (47.3)	15 (44.5)	0.854	41 (95.3)	27 (93.1)	1.00
Female	116 (52.7)	18 (54.5)		2 (4.7)	2 (6.9)	
Tobacco smoking history						
Never	139 (63.2)	20 (60.6)	0.847	8 (18.6)	5 (17.2)	1.00
Ever/current	81 (36.8)	13 (39.4)		35 (81.4)	24 (82.8)	
CEA level (*n* = 395)						
<5.0 IU/mL	183 (83.2)	14 (42.4)	<0.0001	33 (76.7)	20 (69.0)	0.587
≥5.0 IU/mL	37 (16.8)	19 (57.6)		10 (23.3)	9 (31.0)	
Tumor location						
Upper lobe	131 (59.5)	18 (54.6)	0.145	23 (53.5)	14 (48.3)	0.760
Middle lobe	17 (7.7)	3 (9.1)		1 (2.3)	2 (6.9)	
Lower lobe	72 (32.7)	11 (33.3)		15 (34.9)	9 (31.0)	
Middle-lower lobe	0	1 (3.0)		4 (9.3)	4 (13.8)	
Type of surgery						
Lobectomy	197 (89.5)	27 (81.8)	0.236	38 (88.4)	26 (89.7)	0.591
Limited resection	23 (10.5)	6 (18.2)		5 (11.6)	3 (10.3)	
Adjuvant chemotherapy						
No	146 (66.4)	0	<0.0001	20 (46.5)	0	<0.0001
Yes	74 (33.6)	33 (100.0)		23 (53.5)	29 (100.0)	
Tumor size, cm						
≤1.0	27 (12.3)	0	0.015	1 (2.3)	0	1.00
1.0–≤2.0	136 (61.8)	18 (54.5)		17 (39.5)	11 (37.9)	
2.0–≤3.0	57 (25.9)	15 (45.5)		25 (58.2)	18 (62.1)	

*CEA* carcinoembryonic antigen, *IAC* invasive lung adenocarcinoma, *SCC* squamous cell lung cancer

### Clinicopathological data: predicting lymph node metastasis

We performed additional analysis of the association of clinicopathological factors and lymph node involvement using logistic multivariable analysis (Table 3). We found that positive CEA levels, SCC, and tumor sizes larger than 1.0 cm were significantly associated with lymph node involvement (*P* < 0.0001, <0.0001, and 0.048, respectively). Preoperational CEA levels and tumor size were also associated with lymph node involvement in 253 IAC patients (*P* < 0.0001 and 0.023, respectively). However, there was no significant association between any of the clinicopathological characteristics and lymph node involvement in SCC patients.

**Table 3:** Logistic multivariable analyses for the prediction of lymph node-negative and lymph node-positive in pT1 IAC and SCC patients

Factors	Odds ratio	95% Confidence interval	*P* value
IAC and SCC patients			
Gender (male vs. female)	1.333	0.475–3.741	0.580
Preoperative CEA level (<5.0 vs*.* ≥5.0 IU/mL)	3.647	1.908–6.971	<0.0001
Histology (IAC vs*.* SCC)	4.286	2.015–9.116	<0.0001
Tumor size, cm (≤1.0 vs*.* >1.0–≤2.0 vs*.*>2.0–≤3.0)	1.744	1.006–3.024	0.048
IAC patients			
Preoperative CEA level (<5.0 vs*.* ≥5.0 IU/mL)	6.132	2.787–13.490	<0.0001
Tumor size, cm (≤1.0 vs. >1.0–≤2.0 vs. >2.0–≤3.0)	2.227	1.119–4.432	0.023

*CEA* carcinoembryonic antigen, *IAC* invasive lung adenocarcinoma, *SCC* squamous cell lung cancer

### Lymph node involvement, clinicopathological characteristics, and overall patient survival

**Fig. 1:**
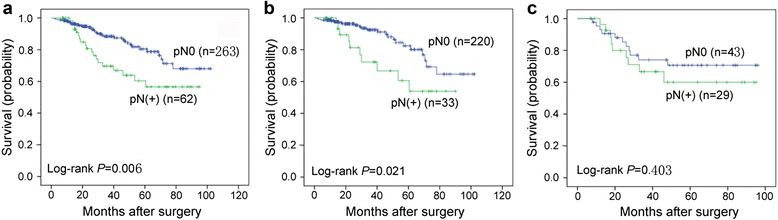
Kaplan-Meier curve analyses of overall survival of patients with pathological T1 IAC or SCC. Overall survival was stratified by lymph node involvement (**a**), IAC lymph node involvement (**b**), or SCC lymph node involvement (**c**)

Median overall survival was 84.8 months for pT1 patients without lymph node involvement, which was significantly longer than the median overall survival of patients with lymph node involvement (66.9 months, *P* = 0.006, Fig. 1a). When we examined the association between lymph node involvement and the overall survival of patients with small-sized IAC tumors or SCC, we found that IAC patients with positive lymph nodes had a median overall survival of 64.1 months. This was a shorter median overall survival when compared with patients without lymph node involvement, a median overall survival of 85.1 months (*P* = 0.021, Fig. 1b). In contrast, no significant difference was observed in overall survival between patients with lymph node-positive SCC and patients with lymph node-negative SCC (67.1 vs. 75.8 months; *P* = 0.403, Fig. 1c). Additionally, multivariable analysis revealed that lymph node involvement was an independent prognostic predictor of overall IAC patient survival (*P* = 0.041, Table 4). However, no statistically significant association was observed between IAC histologic types (lepidic, papillary, etc.) or CT images [pure ground-glass opacity (GGO) vs*.* mixed GGO vs*.* solid nodule] and the prognosis of IAC patients (*P* = 0.716, 0.366). Additionally, no significant association was observed between surgery type (lobectomy vs*.* sublobectomy) and the prognosis of IAC and SCC patients (*P* = 0.166). Additionally, no improvement in the overall survival of patients with a tumor less than 30 mm was observed in patients given adjuvant chemotherapy (*P* = 0.730 and 0.710 in IAC and SCC patients, respectively).

**Table 4:** Univariate and multivariable Cox analyses of overall IAC patient survival

Factors	Univariate analysis			Multivariable analysis		
	Hazard ratio	95% Confidence interval	*P* value	Hazard ratio	95% Confidence interval	*P* value
Gender (male vs. female)	0.495	0.241–1.015	0.055			
Preoperative CEA levels (<5.0 vs. ≥5.0 IU/mL)	2.511	1.245–5.065	0.010	1.865	0.850–4.090	0.120
Lymph node metastasis (absent vs*.* present)	2.360	1.114–5.001	0.025	2.154	1.033–4.493	0.041

*CEA* carcinoembryonic antigen, *IAC* invasive lung adenocarcinoma

Gender (male vs. female, *P* = 0.028) was associated with the overall survival of pT1 patients, but not with IAC or SCC patients (*P* = 0.055 and 1.000, respectively). Univariate analysis showed that preoperational CEA levels were associated with the overall survival of IAC or SCC patients (*P* = 0.010 and 0.021, respectively). However, multivariable analysis did not confirm this result in IAC patients with tumors <30 mm (*P* = 0.120). Lymph node metastasis was an independent survival factor for patients with small-sized IAC (*P* = 0.041, Table 5).

**Table 5:** Univariate and multivariable Cox analyses of overall pT1 IAC and SCC patient survival

Factors	Univariate analysis			Multivariable analysis		
	Hazard ratio	95% Confidence interval	*P* value	Hazard ratio	95% Confidence interval	*P* value
Gender (male vs. female)	0.493	0.263–0.926	0.028	0.525	0.279–0.988	0.046
Preoperative CEA levels (<5.0 vs*.* ≥5.0 IU/mL)	2.777	1.599–4.824	<0.0001	2.455	1.395–4.321	0.002
Lymph node metastasis (absent vs*.* present)	2.189	1.239–3.866	0.007	1.736	0.970–3.109	0.063

*CEA* carcinoembryonic antigen, *IAC* invasive lung adenocarcinoma, *SCC* squamous cell lung cancer

Our data indicated no statistical difference in the survival of SCC patients with or without lymph node involvement. We also analyzed the rate of postoperative chemotherapy in patients with IAC or SCC and found that patients with SCC underwent chemotherapy at a rate of 72.2% (52/72), significantly higher than the rate of 42.3% (107/253) in IAC patients.

## Discussion

In this study, we retrospectively analyzed all primary lung cancer patients who underwent treatment in our hospital between January 2007 and December 2014. Among these patients, we found no lymph node metastases in pathological T1 AIS or MIA patients, which was consistent with previous studies [7, 14]; therefore, we excluded these patients from our current study and chose to investigate the IAC and SCC subtypes of NSCLC [15]. We found that the rate of lymph node involvement was significantly higher in SCC patients when compared with the lymph node involvement in IAC patients. Multiple logistic regression analysis confirmed that SCC was significantly associated with lymph node involvement; however, lymph node involvement was not an independent prognosis factor for pT1 SCC patients. Lymph node-positive IAC was associated with poor overall survival, and lymph node involvement was an independent predictor of overall IAC survival. These results support the notion that additional studies are needed to investigate clinicopathological factors for association with lymph node involvement and NSCLC metastasis.

Previous studies have reported no significant differences in lymph node involvement between IAC and SCC, and that primary lung cancer histology predicted occult mediastinal lymph node metastasis [10, 11]. However, a previous comparative study demonstrated that 12 of 25 of non-adenocarcinoma patients had lymph node involvement [16], and this result is supported by our current study showing that pT1 SCC patients had a higher incidence of lymph node involvement when compared with IAC patients. The variability in the results reported by these different studies may be due to variations in study sample size, as well as the high incidence of IAC patients. Our current study indicated that lymph node involvement was an independent risk factor for overall IAC patient survival, and this result is supported by previous investigations [17, 18]. However, we did not observe that lymph node metastasis was a predictor of poor overall survival in SCC patients. This may be due to the limited number of SCC patients with tumors <30 mm. Furthermore, most SCC patients had a tumor lesion >20 mm and lymph node metastasis; thus, postoperative chemotherapy was given to most SCC patients. Thus, the overall survival of these patients may have been improved by this treatment. Another factor improving patient survival might be the comparatively lower malignancy of SCC relative to IAC, even though these patients had lymph node metastasis.

The systematic dissection of lymph nodes during resection of subcentimeter lung cancer is a controversial procedure. Several studies have reported subcentimeter NSCLCs that were pathologically N1 or N2 [19, 20]. Others have reported no N1 or N2 lymph node involvement in subcentimeter NSCLCs, suggesting that systematic lymph node dissection should be avoided in these cases [11, 21]. In the current study, we found that there was no lymph node metastasis in IAC or SCC patients with tumor lesions less than 1 cm. We also observed that IAC incidence was associated with tumor size. A previous study reported that the incidence of lymph node metastasis increased as tumor size increased [22]. In our study, tumor size was an independent predictor for IAC lymph node metastasis. However, lymph node involvement was independent of tumor size in SCC patients. Tsutani et al. analyzed 100 patients with clinical stage Ia SCC and reported that tumor size did not predict lymph node metastasis [12], and this result was supported by the results of our current study.

The current study observed that the incidence of lymph node involvement was higher in male patients. Furthermore, Cox regression analysis indicated that male patients had significantly poorer overall survival when compared with their female counterparts. This result is consistent with a previous study by Sakurai et al. [23]. Differences in the incidence of lymph node metastasis between male and female patients might be one reason for the different overall survival time for small-sized lung cancer; thus, further study is justified to validate this finding. Preoperative serum CEA levels were associated with IAC lymph node metastasis, but no clinical variables were associated with lymph node involvement in SCC patients. These results are supported by a previous study by Bo et al. [9] that reported that an abnormal CEA titer could predict the rate of lymph node metastasis of T1a lung adenocarcinoma patients, but not of SCC patients, which was reported by Tsutani et al. [12]. Our current study demonstrated that certain clinicopathological factors could be used to predict metastasis of pT1 lung cancer to the lymph nodes; however, the underlying mechanism of this metastasis remains unknown. Our data indicate that pT1 metastasizes to the lymph nodes even though it is an early stage NSCLC. However, further confirmation of this result is needed. Furthermore, our data showed no association between IAC subtypes and CT imaging results and the prognosis of IAC patients, which may be due to the relatively short follow-up period or the small sample size.

Certain limitations of the current study should be considered. The study was conducted solely at a single institution, and the follow-up period was short. A future multicenter study with a longer follow-up period should be performed to confirm the current findings. Additionally, due to the relatively small number of cases available, this study excluded other potentially important histological NSCLC subtypes (e.g., large cell lung cancer) that should be included in future investigations. PET scan was not used in our patients due to availability.

## Conclusions

Patients with lung SCC exhibited a significantly higher rate of lymph node involvement when compared with IAC patients. Additionally, lymph node involvement was an independent prognostic factor for pT1 IAC patients. Thus, our findings suggest that a systematic lymph node dissection should be conducted in patients with IAC larger than 1.0 cm.

## Abbreviations

ADCs: Adenocarcinomas
AIS: Adenocarcinoma in situ
CEA: Carcinoembryonic antigen
CT: Computed tomography
DFS: Disease-free survival
IAC: Invasive adenocarcinoma
IASLC/ATS/ERS: International Association for the Study of Lung Cancer/American Thoracic Society/European Respiratory Society
MIA: Minimally invasive adenocarcinoma
NCCN: National Comprehensive Cancer Network
NSCLC: Non-small cell lung cancer
pT1: Pathological T1
SCC: Squamous cell lung cancer
SUV: Standardized uptake value
TNM: Tumor, node, and metastasis
